# Preserflo-MicroShunt: Postoperative Effects on Endothelial Cell Density and Corneal Thickness

**DOI:** 10.3390/biomedicines13020364

**Published:** 2025-02-05

**Authors:** Sebastian Dierse, Eliane Luisa Esser, Ralph-Laurent Merté, Sami Al-Nawaiseh, Martin Dominik Leclaire, Nicole Eter, Viktoria Constanze Brücher

**Affiliations:** Department of Ophthalmology, University of Munster Medical Center, Albert-Schweitzer-Campus 1, Building D15, 48149 Muenster, Germany

**Keywords:** endothelial cell density, corneal thickness, intraocular pressure, antiglaucoma, MicroShunt

## Abstract

**Background:** The aim of this study was to evaluate the effects of Preserflo implantation on endothelial cell density (ECD), corneal thickness, intraocular pressure (IOP), and the use of antiglaucoma medications over a 12-month follow-up period. **Methods:** A total of 53 eyes from 53 patients undergoing Preserflo implantation were included in this prospective study. ECD, corneal thickness, IOP, and the number of antiglaucoma medications were measured at baseline, 3 months, and 12 months postoperatively. Statistical analysis was performed using paired *t*-tests and Wilcoxon signed-rank tests for non-normally distributed data. **Results:** There was no significant reduction in ECD at 3 months (*p* = 0.695) or 12 months (*p* = 0.229) compared to baseline. However, a significant reduction in corneal thickness was observed at 3 months (*p* = 0.008), with no significant change at 12 months (*p* = 0.118). A significant reduction in IOP was noted at both 3 months (from a preoperative median of 23.5 mmHg to 11.5 mmHg, *p* < 0.001) and 12 months (from 23.5 mmHg to 13.0 mmHg, *p* < 0.001). Additionally, there was a significant decrease in the use of antiglaucoma medications, from a preoperative median of 3.0 medication classes to 0.0 classes at both 3 months (*p* < 0.001) and 12 months (*p* < 0.001). **Conclusions:** Preserflo implantation resulted in a significant reduction in IOP and the need for antiglaucoma medications, with no significant impact on endothelial cell density and corneal thickness after 12 months. These findings suggest that Preserflo implantation is an effective procedure for IOP control and medication reduction, with favorable outcome for corneal health after one year.

## 1. Introduction

Glaucoma is a chronic and progressive optic neuropathy that, in its advanced stages, can significantly impair patients’ quality of life and may lead to blindness [1]. Current treatment strategies focus on the medical or surgical reduction of intraocular pressure (IOP) [2]. Among surgical treatments, trabeculectomy is considered the gold standard. Although this is an established procedure, it is not without potential, sometimes serious, complications [3]. This has driven the search for alternatives through procedures that potentially carry fewer risks of complications.

The term minimally invasive glaucoma surgery (MIGS) encompasses various micro-surgical procedures designed to facilitate aqueous humor outflow. More recently, the terminology has evolved to include the concept of less invasive glaucoma surgery (LIGS) or minimally invasive bleb surgery (MIBS). The Preserflo MicroShunt (Santen, Osaka, Japan) is categorized as a LIGS/MIBS device and is a subconjunctival implant placed via an ab externo approach. It was developed and approved for the treatment of patients with primary open-angle glaucoma (POAG) [4]. The MicroShunt is made of polystyrene-block-isobutylene-block-styrene (SIBS), a biostable thermoplastic elastomer that has demonstrated biocompatibility and long-term stability [5]. The device measures 8.5 mm in length, with an outer diameter of 350 μm and a lumen of 70 μm. A 1.1 mm wing prevents dislocation and peri-tubular leakage. Early studies report promising success rates with a manageable risk profile [6,7,8,9,10].

Although the implant has a smaller diameter compared to other glaucoma drainage devices (e.g., Paul, Ahmed, Baerveldt, Molteno), the presence of a foreign body in the anterior chamber may potentially affect the corneal endothelial cell density (ECD). The extent of endothelial cell loss varies depending on the implant’s proximity to the corneal endothelium [11,12,13]. Due to data from the COMPASS XT study, which demonstrated a statistically significant 20.4% reduction in endothelial cell density (95% CI: −23.5%, −17.5%) in patients with a CyPass MicroStent over a 5-year follow-up period [14], the CyPass MicroStent was withdrawn from the market in 2018.

Currently, there is limited information regarding the medium- and long-term effects of the Preserflo MicroShunt on corneal endothelial cells and corneal thickness.

The aim of the present study is to analyze the changes in ECD and corneal thickness in the first year after the implantation of a Preserflo MicroShunt.

## 2. Materials and Methods

### 2.1. Study Design

This is a non-randomized, prospective, single-arm clinical study with a follow-up period of 12 months. The measurements were performed prior to the planned surgical procedure, at month three, and at month twelve after the operation. The study received approval from the Ethics Committee of the University of Muenster and adhered to the principles outlined in the Declaration of Helsinki (2023-161-f-S) on 29 September 2023. Informed consent was obtained from all participants after a thorough explanation of the examinations.

### 2.2. Inclusion and Exclusion Criteria

Patients included in the study had a diagnosis of primary chronic open-angle glaucoma (POAG), pseudoexfoliation glaucoma (PXG), normal tension glaucoma (NTG), or pigment dispersion glaucoma (PGD) (Table 1).

Participants underwent a Preserflo MicroShunt implantation, with both preoperative and postoperative endothelial cell density (ECD) measurements. Exclusion criteria included previous filtering glaucoma surgeries, inflammatory eye diseases from the uveitis spectrum, corneal diseases, especially endothelial dystrophies, or previous keratoplasty. Additional pressure-lowering surgical interventions during the 12-month follow-up period led to subsequent exclusion from the study. All patients underwent slit-lamp biomicroscopy and fundus examination. Intraocular pressure (IOP) was measured using Goldmann applanation tonometry, and visual acuity was determined.

### 2.3. Equipment / Definitions and Statistical Analysis

ECD was measured using the Konan CellCheck SL (Konan Medical, Marconi, STEA Irvine, CA, USA) with software version 25.1.0. The cell analysis was based on the following mathematical equation: CD = number of analyzed cells/area of selected, analyzed cells. The cell images were saved in RGB JPEG format, 24-bit, 304 (H) × 446 (W). Anterior segment images were also saved in RGB JPEG format, 24-bit, 640 (H) × 480 (W).

Corneal thickness was measured using the Pentacam (Oculus Optikgeräte GmbH, Germany). Visual field testing at 30° was performed with the Zeiss Humphrey Field Analyzer 3, version 1.5.3.714 (Carl Zeiss AG, Oberkochen, Germany). Optical coherence tomography (OCT) of the optic nerve head with retinal nerve fiber layer (RNFL) thickness measurements was performed using the Spectralis OCT Spec-KT-06560 (Heidelberg Engineering GmbH, Heidelberg, Germany).

The preoperative timepoint was defined as the most recent examination before surgery, which occurred no more than 4 weeks prior to the procedure. Postoperative measurements were taken 3 and 12 months after the Preserflo MicroShunt implantation. Visual acuity was recorded in logMAR. Ocular hypotony was defined as an IOP < 5 mmHg.

Statistical analysis was performed using SPSS Statistics for Windows, version 29.0.0 (IBM Corp., Armonk, NY, USA). Data were analyzed using the Wilcoxon rank test. Results are presented as median and interquartile ranges. Statistical significance was set at *p*-values ≤ 0.05.

### 2.4. Endothelial Cell Density Measurement

The measurements were performed by specially trained medical photographers from the imaging department, with the same individual conducting all measurements. The device automatically compared the collected data during follow-up examinations to ensure that anatomically identical measurement points were used.

### 2.5. Surgical Technique

After opening the conjunctiva and Tenon’s capsule, the Tenon’s capsule was dissected from the sclera to create a subconjunctival pocket behind the limbus. Intraoperatively, the subconjunctival space was treated for three minutes with three sponges soaked in 0.2 mg/mL mitomycin C (MMC). The incision site for the scleral tunnel was marked 4 mm from the surgical limbus towards the sclera, and a 2 mm long scleral tunnel was created with a 1.1 mm lance. This tunnel was then extended using a 25-gauge needle to form a transscleral tunnel into the anterior chamber (AC) at the level of the trabecular meshwork. The Preserflo MicroShunt was introduced into the anterior chamber using tying forceps (Geuder, Heidelberg, Germany) and advanced until the implant’s fins rested within the sclera. Finally, the Tenon’s capsule and conjunctiva were sutured.

Postoperatively, antibiotic and corticosteroid eye drops were administered, with the corticosteroids tapered over a period of six months. Depending on IOP, scarring tendencies, and bleb configuration, sub-Tenon 5-fluorouracil (5-FU) injections were given within the first four weeks.

## 3. Results

### 3.1. Patient Cohort

A total of 53 eyes from 53 patients were included in the study. One eye of a patient was excluded from the analysis during the study due to the need for a trabeculectomy after 10 months, which was performed as a result of insufficient intraocular pressure reduction and anterior chamber hemorrhage. The preoperative demographic data are presented in Table 1.

The average follow-up interval after 3 months was a median of 3.52 (IQR 2.99;5.03) and, after 12 months, was a median of 12.03 (IQR: 10.06;13.62).

### 3.2. Endothelial Cell Density (ECD)/Corneal Thickness

**Figure 1:**
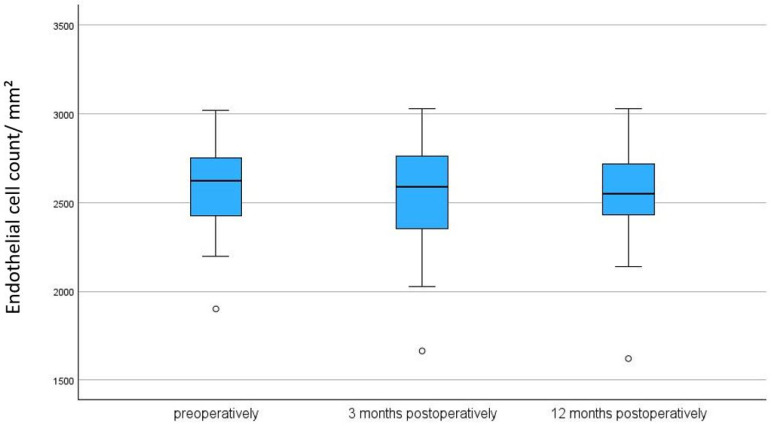
Endothelial cell count preoperatively, after 3, and after 12 months following Preserflo implantation. *p* = 0.229. The box plot diagram shows the median, 25th and 75th percentiles, and the minimum and maximum values (whiskers). mm^2^ = square millimeters, ECD = endothelial cell density. ° represent outliers.

**Figure 2:**
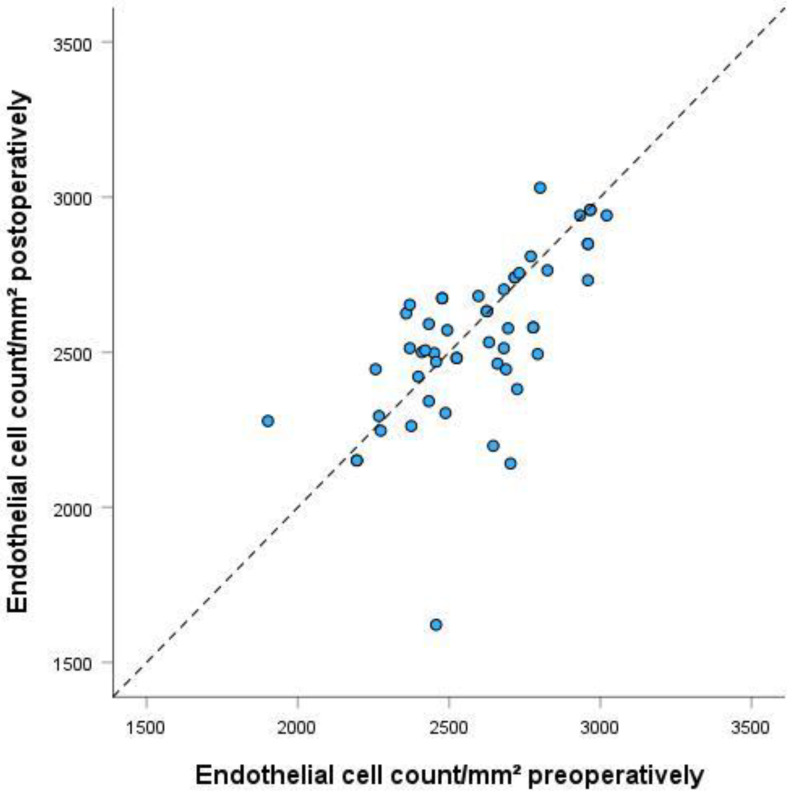
Endothelial cell count preoperatively and 12 months after Preserflo implantation. *p* = 0.229. The scatter plot shows the endothelial cell counts pre- and postoperatively per mm^2^. ° represent outliers.

**Figure 3:**
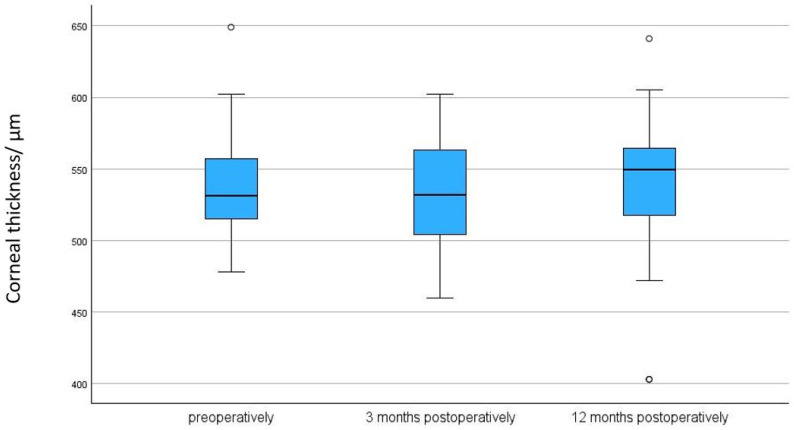
Corneal thickness preoperatively, after 3, and after 12 months following Preserflo implantation. *p* = 0.118. The box plot diagram shows the median, 25th and 75th percentiles, and the minimum and maximum values (whiskers). µm = micrometers, CT = corneal thickness. ° represent outliers.

There was no significant reduction in ECD after 3 months (*p* = 0.695) and after 12 months compared to baseline (*p* = 0.229) (see Table 2 and Table 3, and Figure 1 and Figure 2). There was a significant reduction in corneal thickness after 3 months (*p* = 0.008) and no significant reduction after 12 months compared to baseline (*p* = 0.118) (see Table 2 and Table 3, and Figure 3).

### 3.3. Intraocular Pressure (IOP)

**Figure 4:**
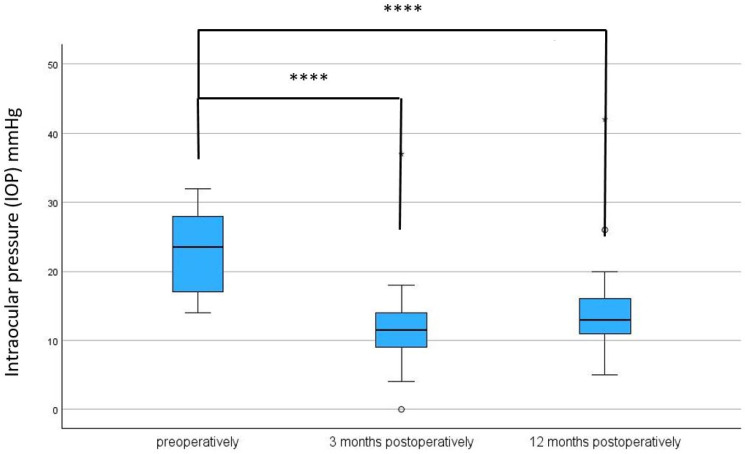
Intraocular pressure preoperatively, after 3, and after 12 months following Preserflo implantation. **** = *p* < 0.001. The box plot diagram shows the median, 25th and 75th percentiles, and the minimum and maximum values (whiskers). IOP = intraocular pressure, mmHg = millimeters of mercury. ° and * represent outliers.

After a 3-month and a 12-month follow-up period, a significant reduction in IOP was observed, from a preoperative median of 23.5 (IQR: 17;28) mmHg to a postoperative median of 11.50 (IQR: 5.6;14) mmHg (*p* < 0.001) at 3 months and 13.00 (IQR: 5.9;16) mmHg (*p* < 0.001) at 12 months (see Table 2 and Table 3, and Figure 4).

### 3.4. Antiglaucoma Medications

**Figure 5:**
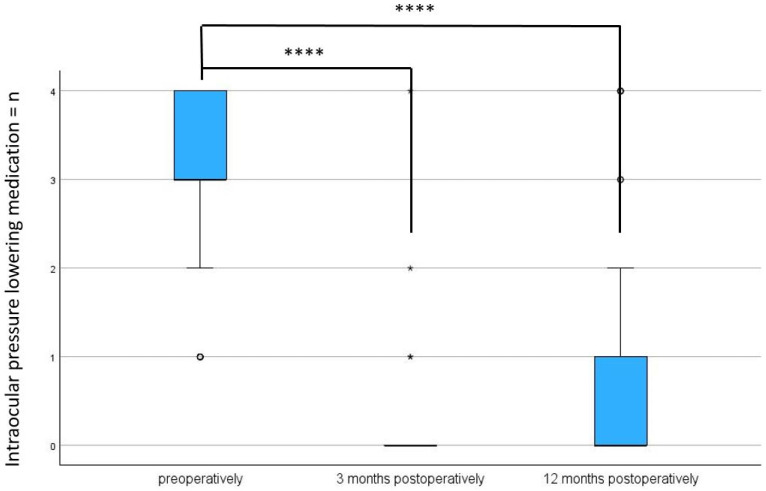
Reduction in IOP-lowering medications preoperatively, after 3, and after 12 months postoperation. **** = *p* < 0.001. The box plot diagram shows the median, 25th and 75th percentiles, and the minimum and maximum values (whiskers). *n* = number, IOP = intraocular pressure. ° and * represent outliers.

There was also a statistically significant reduction in antiglaucoma medications, from a preoperative median of 3.00 (IQR: 3;4) medication classes to a postoperative median of 0 (IQR: 0;1) (*p* < 0.001) after 3 months and a median of 0 (IQR: 0;1) (*p* < 0.001) after 12 months (see Table 2 and Table 3 and Figure 5).

### 3.5. Postoperative Complications/Implant Position

One eye developed an anterior chamber hemorrhage (1.5 mm hyphema) on the second postoperative day, which resolved rapidly with topical steroid therapy. Six months after the implantation, this eye experienced a refractory IOP increase, reaching levels between 25 and 30 mmHg. A trabeculectomy was performed, and following this, IOP values stabilized within the target range (12-month follow-up period). This eye exhibited 21% endothelial cell loss 12 months after the initial Preserflo implantation (10 months post-trabeculectomy), from 2703 cells/mm^2^ preoperatively to 2141 cells/mm^2^. This patient was excluded from the study.

The position of the Preserflo MicroShunt was evaluated using anterior segment OCT in all eyes. At 12 months postoperatively, none of the eyes (*n* = 52) showed contact between the shunt and either the iris or cornea. Ocular hypotony within the first week was observed in 18 eyes, of which 8 showed peripheral choroidal effusion. The condition resolved completely following topical and systemic steroid administration.

## 4. Discussion

Regarding postoperative changes in corneal endothelial cells, Lass et al. demonstrated a significant decrease in endothelial cells following the implantation of a CyPass Micro-Stent [13], which heightened awareness of endothelial cell density (ECD) loss after the insertion of intraocular pressure (IOP)-lowering implants especially when placed into the anterior chamber. To date, there are limited data on ECD changes following Preserflo implantation. The Preserflo is increasingly used in clinical practice, and with the rising number of surgeries involving Preserflo implants, further data on ECD changes would be valuable.

The only larger study that considered potential ECD changes was conducted by Baker et al. [10]. Baker et al. investigated the efficacy of the Preserflo compared to trabeculectomy in a two-year prospective, randomized study. Their published one-year results showed that the Preserflo group (*n* = 362) experienced only a slight endothelial cell loss of −5.2%. The mean endothelial cell density decreased from 2310.7 ± 359.6 cells/mm^2^ at baseline (*n* = 394) to 2199.7 ± 443.5 cells/mm^2^ after one year (*n* = 362). This figure was purely descriptive and not tested for statistical significance. One patient exhibited a loss of 9.4%, which the authors attributed to the proximity of the implant to the cornea [10]. In contrast to our study, only patients with primary open-angle glaucoma were included, and no consideration was given to pre-existing corneal disease. The only exclusion criterion related to the cornea was a history of previous corneal surgery.

A smaller population was studied by Steindor et al., who included 14 eyes from 12 patients. They also demonstrated no significant endothelial cell loss (preoperative ECD: 2074 ± 706.6 cells/mm^2^; postoperative: 2029 ± 742.3 cells/mm^2^; *p* = 0.42) [15]. With a similar average age of 68 ± 9.8 years, their patient population was comparable to that of our study. The minimum follow-up period was 12 months, with an average of 20 ± 2.7 months. Our study design included a planned follow-up period of 3 and 12 months, with an average follow-up duration of 13.66 months (median: 13.64 months) at the 12-month timepoint, ensuring a more stable follow-up and contributing to the validity of the data.

Steindor et al. found no correlation between IOP or ECD and the position of the Preserflo shunt in the anterior chamber [15]. In contrast, Ibarz-Barberá et al. reported a significant endothelial cell loss of 7.4% in the first 12 months (*p* = 0.04) and noted a positive correlation between the shunt position and cell loss. A loss of 18% was observed when the distance between the shunt and the endothelium was less than 200 µm [16]. Notably, 30.4% of the patients underwent combined surgery with phacoemulsification. A subgroup analysis revealed that ECD loss was higher in combined procedures than in standalone surgeries (Month 1: 140 ± 110 vs. 118 ± 181), although this was not statistically significant [16]. Cataract surgery, therefore, should be considered as a potential cause for endothelial cell loss in such cases.

In our study, the overall cohort also showed no significant ECD reduction. Only one eye exhibited 21% endothelial cell loss 12 months after implantation. On the first postoperative day, this eye displayed a 1.5 mm hyphema upon slit-lamp examination. The condition improved with topical steroid therapy. As the implant did not contact the endothelium, we attribute the endothelial cell loss to the potential toxicity of erythrocytes on the endothelium [17,18,19]. Six months postoperatively, the patient developed a flattened bleb with insufficient filtration, leading to a rise in IOP to 25 mmHg, which necessitated a trabeculectomy.

This is consistent with the prospective study by Gassel et al., which evaluated 62 eyes from 55 patients with open-angle glaucoma over a two-year period following Preserflo MicroShunt implantation. The study demonstrated a significant reduction in intraocular pressure, from 29.6 ± 8.3 mmHg preoperatively to 13.0 ± 4.3 mmHg at 24 months, with no significant loss in corneal endothelial cell density and stable best-corrected visual acuity [20].

Chamard et al. reported endothelial cell loss in 2 of 16 eyes following Preserflo MicroShunt implantation [21]. In the first case, the affected eye had an endothelial cell count of 549 cells/mm^2^ five years post-implantation (fellow eye: 2559 cells/mm^2^). An OCT scan revealed a positional change of the implant with newly developed corneal contact, which was considered the cause of the endothelial cell loss, leading to implant removal. In the second case, a reduced endothelial cell count was noted five years post-implantation compared to the fellow eye (1139 cells/mm^2^ vs. 2366 cells/mm^2^). OCT showed a hyperreflective structure surrounding the implant, which the authors attributed to a localized inflammatory reaction as the cause of the endothelial cell loss. No difference in corneal thickness was observed in either eye with endothelial cell loss. One limitation of this study is that no pre-implantation endothelial cell measurements were taken, and instead, the fellow eye was used as a control reference. For the remaining 10 eyes (4 were not followed up with), no significant difference between implanted and fellow eyes was observed, leading the authors to conclude that the endothelial cell loss was related to the MicroShunt implantation [22].

Riss et al. described the results of a retrospective analysis from 2015 with a one-year follow-up after MicroShunt implantation. They reported effective pressure reduction and a decrease in the need for antiglaucoma therapy [14]. The average visual acuity of 0.39 logMAR (Table 1) suggests that invasive procedures like the Preserflo are often reserved for later stages of the disease, typically after other therapies have failed.

The efficacy and benefits of the Preserflo have been well documented in the literature, with numerous studies reporting similar outcomes depending on the patient population [6,8,13,22,23,24]. A larger study published in 2016 by Battle et al. reported long-term results on the efficacy of the implant [25]. Over a three-year follow-up of 23 eyes, the qualified success rate (IOP ≤ 14 mmHg and IOP reduction ≥ 20%) ranged between 91% (Year 2) and 100% (Year 1). The mean medicated IOP decreased from 23.8 ± 5.3 mmHg to 10.7 ± 2.8, 11.9 ± 3.7, and 10.7 ± 3.5 mmHg. The average number of glaucoma medications per patient was reduced from 2.4 ± 0.9 to 0.3 ± 0.8, 0.4 ± 1.0, and 0.7 ± 1.1, respectively. However, other studies do not report such high success rates, which may be explained by the small sample size (*n* = 23). Additionally, it should be noted that Battle et al.‘s study exclusively included eyes with primary open-angle glaucoma [25].

Other authors report success rates (complete success) between 61% and 76% [6,8,22,26,27]. In another study, Battle et al. reported follow-up results for the same cohort over five years. IOP decreased from 23.8 ± 5.3 mmHg with 2.4 ± 1.0 medications to 12.4 ± 6.5 mmHg with 0.8 ± 1.3 medications at the fifth year [9]. Steindor et al. reported a decrease in IOP by 8.2 mmHg, which is a smaller reduction compared to our cohort. This difference could be due to the heterogeneity of the postoperative periods or a lower preoperative IOP baseline.

Our data, however, show comparable results in terms of pressure reduction and reduction in topical medications. Twelve months after Preserflo MicroShunt implantation, we observed a significant IOP reduction from a preoperative median of 23.5 (IQR: 17–28) mmHg to a postoperative median of 13.00 (IQR: 11–16) mmHg (*p* < 0.001). The same was true for the reduction in the number of antiglaucoma medications. Our findings confirm the effectiveness of the Preserflo in lowering IOP and reducing the need for antiglaucoma medications.

Based on our results, we can conclude that there is alignment with the outcomes reported in the literature. No significant endothelial cell loss was observed after 3 and 12 months post-implantation.

Our cohort showed a significant reduction in corneal thickness after three months (*p* = 0.008). We suspect that, preoperatively, the cornea swells slightly due to elevated intraocular pressure. Following the pressure reduction achieved by the Preserflo operation, the cornea likely undergoes significant deswelling in the early postoperative phase [28]. Also, the reduction might be attributed to postoperative factors such as inflammation, changes in intraocular pressure (IOP), and exposure to mitomycin-C (MMC), which is commonly used during surgery to prevent scarring but may impact corneal health [28]. No significant changes are observed at 12 months, which is consistent with the findings of Chamard et al. [21] and Steindort et al. [15].

In conclusion, this prospective study suggests that the Preserflo MicroShunt provides effective IOP reduction with significantly reduced medication needs and a good safety profile regarding endothelial cell count after one year. Further prospective studies are needed to determine the long-term success rates and impact on endothelial cell density.

## Figures and Tables

**Table 1:** Demographic data of the study population.

	Study Group (MD ± SD)
Number of eyes (*n*)	52
Gender (female/male)	25/27
Age (years)	67.88 ± 14.28
Visual field (MD)	14.37 ± 9.88
RNFL OCT	50.14 ± 14.22
Visual acuity (logMAR)	0.39 ± 0.32
POAG/PXG/NTG/PGD	34/10/2/6

*n* = number, MD = mean deviation, SD = standard deviation, POAG = primary open angle glaucoma, PXG = pseudoexfoliation glaucoma, NTG = normal tension glaucoma, PGD = pigment dispersion glaucoma.

**Table 2:** Postoperative results after 3 and 12 months.

Result	Baseline	After 3 Months	*p*-Value	After 12 Months	*p*-Value
ECD/mm^2^	2625 (2424;2760)	2591 (2350;2766)	0.695	2551 (2427;2724)	0.229
CT/µm	531 (515;5570)	532 (502;563)	**0.008**	549 (517.25;564.75)	0.118
IOP/mmHg	23.5 (17;28)	11.5 (5.6;14)	**<0.001**	13 (5.9;16)	**<0.001**
AM/*n*	3 (3;4)	0 (0;0)	**<0.001**	0 (0;1)	**<0.001**

ECD = endothelial cell count, CT = corneal thickness, IOP = intraocular pressure, AM = antiglaucoma medication, mm^2^ = square millimeter, µm = micrometer, mmHg = millimeters of mercury, *n* = number. Continuous variables are reported as median (25% quantile; 75% quantile). *p*-values are from pairwise comparisons of baseline and three month and twelve month response values using two-sided exact Wilcoxon signed-rank tests. Bold: *p*-values ≤ 0.05.

**Table 3:** Changes between preoperatively (t_0_) and 3 months (t_3_) (Δt_3_–t_0_) and preoperatively (t_0_) and 12 months (t_12_) (Δt_12_–t_0_).

Result	Δt_3_–t_0_	*p*-Value	Δt_12_–t_0_	*p*-Value
ECD/mm^2^	0 (−132;110)	0.695	−8 (−116.75;69.25)	0.229
CT/µm	−10 (−22.5;2)	**0.008**	5.5 (−11.5;20)	0.118
IOP/mmHg	−9.5 (−16;−5)	**<0.001**	−9 (−13.75;−3.5)	**<0.001**
AM/*n*	−3 (−4;−2)	**<0.001**	−3 (−4;−1)	**<0.001**

ECD = endothelial cell count, CT = corneal thickness, IOP = intraocular pressure, AM = antiglaucoma medication, mm^2^ = square millimeter, µm = micrometer, mmHg = millimeters of mercury, *n* = number. Continuous variables are reported as median (25% quantile; 75% quantile). *p*-values are from pairwise comparisons of baseline and three months resp twelve months values using two-sided exact Wilcoxon signed-rank tests. Bold: *p*-values ≤ 0.05.

## Data Availability

The raw data supporting the conclusions of this article will be made available by the authors on request.
